# Differences in Pathogenicity and Vaccine Resistance Discovered between Two Epidemic Strains of Marek’s Disease Virus in China

**DOI:** 10.3390/v15040945

**Published:** 2023-04-11

**Authors:** Zheng-Hao Yu, Yan-Ping Zhang, Xing-Ge Lan, Ya-Nan Wang, Rong-Rong Guo, Kai Li, Li Gao, Xiao-Le Qi, Hong-Yu Cui, Xiao-Mei Wang, Yu-Long Gao, Chang-Jun Liu

**Affiliations:** Avian Immunosuppressive Diseases Division, State Key Laboratory for Animal Disease Control and Prevention, Harbin Veterinary Research Institute of the Chinese Academy of Agricultural Sciences, Harbin 150069, China

**Keywords:** IFN-β, IFN-γ, immunosuppression, Marek’s disease virus, pathogenicity, vaccination

## Abstract

Despite highly effective vaccines, Marek’s disease (MD) causes great economic loss to the poultry industry annually, largely due to the continuous emergence of new MD virus (MDV) strains. To explore the pathogenic characteristics of newly emerged MDV strains, we selected two strains (AH/1807 and DH/18) with clinically different pathotypes. We studied each strain’s infection process and pathogenicity and observed differences in immunosuppression and vaccine resistance. Specific pathogen-free chickens, unvaccinated or vaccinated with CVI988, were challenged with AH/1807 or DH/18. Both infections induced MD damage; however, differences were observed in terms of mortality (AH/1807: 77.8%, DH/18: 50%) and tumor rates (AH/1807: 50%, DH/18: 33.3%). The immune protection indices of the vaccine also differed (AH/1807: 94.1, DH/18: 61.1). Additionally, while both strains caused interferon-β and interferon-γ expression to decline, DH/18 infection caused stronger immunosuppression than AH/1807. This inhibition persisted even after vaccination, leading to increased replication of DH/18 that ultimately broke through vaccine immune protection. These results indicate that both strains have different characteristics, and that strains such as DH/18, which cause weaker pathogenic damage but can break through vaccine immune protection, require further attention. Our findings increase the understanding of the differences between epidemic strains and factors underlying MD vaccination failure in China.

## 1. Introduction

Marek’s disease (MD), a fatal disease in poultry, is one of the most serious threats to commercial chicken production [1] and is characterized by T-cell lymphoma and immunosuppression. MD is caused by Marek’s disease virus (MDV), a highly contagious cell-associated alpha herpesvirus [2]. MDV continues to evolve, inducing varying degrees of lymphoproliferative lesions in chickens [3]. Witter et al. classified MDV strains as mild (m), virulent (v), very virulent (vv), and very virulent plus (vv+) according to their pathogenic characteristics and the ability of the vaccine to protect against them [4]. However, MDV strains are evolving and rapidly taking on new features, and the current vaccine cannot provide effective protection against all strains [5]. In order to effectively control MDV, the characteristics of new MDV strains, including their immune evasion and resistance abilities, must be identified.

Vaccination is the primary approach used to control MD in chickens [6], and the widespread use of vaccines has achieved good results. However, due to its continued evolution, MDV remains a serious threat to poultry and causes substantial economic losses worldwide annually [7]. Current vaccines cannot induce sterile immunity, allowing MDV to exist in vaccinated hosts and be released into the environment [8]. Thus, current vaccines can lead to MDV evolution [9]. According to our latest research findings, the CVI988 strain, upon which the vaccine is based, as well as a virulent strain have undergone natural recombination, resulting in the evolution of strains with stronger replication abilities than the original CVI988 strain [10]. We have previously reported that co-infection with multiple MDV strains may also prompt strain recombination, leading to MDV strain evolution [11]. Additionally, by analyzing the whole genome, we revealed that MDV is indeed evolving at a high rate [12], with the above being the most important reasons driving its evolution.

The continuous evolution of MDV is the main factor leading to vaccine immunity failure. In recent years, some MDV strains have been found to have new characteristics. For example, LZ1309 infection exhibits a decrease in observed tumor incidence, but a longer latent infection period and increased likelihood of causing severe immunosuppression [13]. In contrast, BS/15 infection exhibits higher tumor rates, prolonged survival, and reduced immunodeficiency [14]. The emergence of these new MDV strains in the field has been suggested as one of the main causes of vaccination failure, resulting in new threats and greater losses to the poultry industry [13,15]. The potential differences in pathogenicity, replication capacity, and immune response among different circulating MDV strains, and whether any such differences influence their relative abilities to induce immune failure, remains unclear. Therefore, the isolation and culture of MDV field strains is essential for monitoring the differences in these strains and their potential correlations with incidences of immune failure.

In this study, we isolated two MDV field strains with different clinical features, AH/1807 and DH/18, from layer and broiler flocks, respectively. AH/1807 can cause common MD clinical signs such as deaths of chickens and organic tumors. However, DH/18 only causes very severe folliculitis and defoliation of paw pads in infected chickens, without causing deaths and organic tumors. Differences in the infection processes and pathogenicity of AH/1807 and DH/18 were studied in specific pathogen-free (SPF) chickens. Differences in immunity against the two strains were also explored. In summary, this study analyzed the differences between two epidemic strains of MDV and the factors influencing immune failure to provide guidance for MD prevention and control.

## 2. Materials and Methods

### 2.1. Collection of Clinical Samples

Feather pulp samples were collected from two separate MD outbreaks on chicken farms in China. The AH/1807 strain derived from the first outbreak in Anhui province of China. It occurred in a commercial laying hen farm, with morbidity and mortality peaking at 90 days of age. AH/1807 can cause the death of chickens and organic tumors. The DH/18 strain derived from the second outbreak in Jinlin province of China. It occurred on a large broiler farm with an incidence of about 20% and no recorded deaths, but very serious folliculitis and cracking of the paw pads in infected chickens were recorded. Feather pulps were collected from all chickens with suspected MD for later molecular diagnosis and viral isolation.

### 2.2. Viral Isolation and Identification

Viral isolation from feather pulp samples was performed as described in a previous study [16]. The (Marek’s EcoRI-Q) *meq* gene and genomic 132-base pair repeat sequence (132 bpr) of MDV were amplified using a polymerase chain reaction (PCR)-based method for viral identification. These two genes can be used to clearly distinguish wild-type strains from vaccine strains [17]. The GA strain, an MDV virulent strain from the United States, was used as positive control in this experiment. Cell cultures of viruses were used as DNA templates for PCR amplification. The PCR primers used for MDV strain identification are used as in the previous study [14].

### 2.3. Screening of Adventitious Agents

Since the occurrence of MDV on chicken farms is often accompanied by many other tumorous or tumor-related diseases [18,19,20], the screening of these adventitious pathogens is essential to ensure the purity of identification of MDV strains. As such, we used methods outlined in previous studies [21,22]. PCR and enzyme-linked immunosorbent assays (ELISAs) were used to detect avian leukosis virus (ALV). A method of PCR and IFA were performed for reticuloendotheliosis virus (REV). Additionally, PCR was used to detect chicken infectious anemia virus (CIAV) in our samples.

### 2.4. Pathogenic Processes and Virulence Studies

AH/1807 and DH/18 were used as the challenge strains. The CVI988 vaccine from commercial vaccine (CVI988 /Rispens strain) was used as the vaccine efficacy evaluation strain, because it confers the highest protection against MDV among all commercially available vaccines [23]. In total, 180 one-day-old SPF White Leghorn chickens were obtained from the Experimental Animal Center (EAC) at the Harbin Veterinary Research Institute of the Chinese Academy of Agricultural Sciences, Harbin, China. Additionally, they are heterozygous MHC-B haplotypes. Chickens were randomly divided into nine groups (see Table 1 for details) and kept in a negative-pressure isolator with adequate water and food supply and a comfortable environment.

Vaccination was performed at the age of one day. An immune dose of 200 µL of diluent containing 2000 plaque-forming units (PFUs) was administered to each chicken in groups 4, 5, 8, and 9 via intraperitoneal injection. Chickens in all other groups received 200 µL of diluent injected in the same manner. On day 7 post-vaccination, an MDV challenge was performed via intraperitoneal injection with 1000 PFU of one of the two isolated strains of MDV (groups 2, 3, 4, and 5: AH/1807; groups 6, 7, 8, and 9: DH/18) suspended in 200 µL diluent. Chickens from group 1 received the same amount of diluent in the same manner, but without the PFU, thus serving as controls.

The health status of the group 1 chickens was observed every day to ensure the establishment of the healthy control group for accurately determining the incidence of MD. Chickens in groups 3, 5, 7, and 9 were observed daily for clinical signs of MD. The MD status of the experimental animals was estimated by monitoring for early mortality syndrome, immune organ damage, and/or tumor formation. Total chicken deaths in each group were recorded throughout the experimental period for later survival analysis (groups 3, 5, 7, and 9). Three chickens were randomly selected from the control and challenged groups (groups 2, 4, 6, and 8) for postmortem examination at 4-, 7-, 14-, 21-, and 28-days post challenge (dpc), and body weight and weight of the immune organs (thymus, spleen, and bursa) were measured at these times. The viral loads in the spleens of chickens from the challenged groups (groups 2, 4, 6, and 8) were analyzed dynamically. During the experimental period, treatments were applied using gentle movements to avoid frightening the animals, and all chickens were humanely euthanized and immediately autopsied at the end of the experiment (72 dpc). These experiments have been performed in a single-blind manner.

### 2.5. DNA and RNA Extraction and Quantitative Real-Time Polymerase Chain Reaction 

The collected spleens were separately homogenized. DNA was extracted using the AxyPrep Body Fluid Viral DNA/RNA Miniprep Kit (Corning Life Sciences Co., Ltd., Suzhou, China), according to the manufacturer’s instructions. To quantify the in vivo replication of the two strains, the MDV *meq* gene was used as a quantitative real-time polymerase chain reaction (qRT-PCR) target gene in the MDV genome, while the chicken ovotransferrin (*ovo*) gene was used as an internal reference gene in the chicken cell genome. Total RNA was extracted from the collected spleen samples, and cDNA was then extracted from the total RNA using the BioRT Master HiSensi cDNA First Stand Synthesis Kit (BIOER Co., Ltd., Hangzhou, China) and stored at −20 °C. Expressions of the interferons (*IFNs*) *IFN-β* and *IFN-γ* were measured as indicators of immune response. The qRT-PCR and measurement of *IFN-β* and *IFN-γ* were performed as described in previous studies [24]. All primers and probes used for the qRT-PCR are listed in Table 2.

### 2.6. Histopathological Examination

Samples of tissues or organs were collected from deceased and diseased chickens for histopathological examination. Samples were fixed in 10% formaldehyde, routinely processed, embedded in paraffin wax, mounted on glass slides, and stained with hematoxylin and eosin (HE). Image Pro Plus 6.0 software (Media Cybernetics Inc., Rockville, MD, USA) with “measurement staining” capabilities was then used to analyze histopathological findings based on optical density and percentage area.

### 2.7. Sequence Alignment and Phylogenic Analysis

The DNA of AH/1807 and DH/18 as PCR templates was extracted from inoculated duck embryo fibroblasts culture samples. PCR amplification was performed as previously described [17]. Primers of *meq* were used the same as previous study [14]. PCR products were purified and ligated to the pMD 18-T vector (Takara Biotechnology) [25]. Regarding sequencing, alignment and phylogenic analysis are shown as previously reported [11]. The *meq* gene sequences of AH/1807 and DH/18 can be found in Supplementary Table S1. Additionally, sequences of AH/1807 and DH/18 were compared with those of the published strains from GenBank database.

### 2.8. Statistical Analysis

The vaccine protective index (PI) for each strain was calculated using the following formula: PI = [(%MD in unvaccinated chickens − %MD in vaccinated chickens)]/%MD in unvaccinated chickens) × 100, as described in a previous study [26]. The absolute number of MDV genome copies per million cells in the spleen was calculated using the following formula: normalized viral load = log_10_ ((MDV genome copy number/chicken genome copy number) × 10^6^); and expression of *IFNs* in the spleen was calculated using the following formula: log_2_ fold change(mRNA) = log_2_ (*IFN* copy number/*28S* copy number). Viral load, body weight, weight of each immune organ/weight of chicken, survival analysis, and log_2_ fold change (mRNA) data were analyzed using GraphPad Prism (version 7.02; GraphPad Software, Inc., San Diego, CA, USA). Data for the two MDV strains were compared at each time point. Data analysis was performed using a two-way analysis of variance. The differences were considered statistically significant at * *p* < 0.05, ** *p* < 0.01, *** *p* < 0.001, and **** *p* < 0.0001. All data are presented as means ± standard deviations (SDs).

## 3. Results

### 3.1. Two MDV Strains Isolated in China

**Figure 1:**
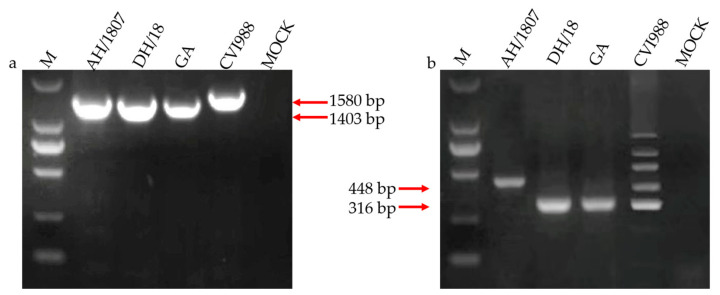
Detection of Marek’s disease virus (MDV) by polymerase chain reaction (PCR). DNA of MDV-infected duck embryo fibroblasts (DEFs) was used as the template for PCR amplification. (**a**) PCR amplification of the Marek’s EcoRI-Q (*meq*) gene of MDV. The PCR products of AH/1807 and DH/18 were 1,403 base pairs (bp) long; (**b**) PCR amplification of the 132-bp repeat (bpr) of MDV. The PCR product of AH/1807 132-bpr was 448 bp long with a copy number of 3, while the PCR product length for DH/18 was 316 bp with a copy number of 2. (M) DL 2000 DNA Marker; CVI988: Culture of CVI988 vaccine stain infected duck cells; GA: Culture of MDV strain GA-infected duck cells; MOCK: Culture of uninfected duck cells.

Two unique MDV strains, AH/1807 and DH/18, were successfully isolated from infected layers and broilers, respectively. PCR detection of MDV using the *meq* (Figure 1a) and 132-bpr (Figure 1b) genes revealed that both strains were of the wild type, with characteristics that differed from those of the vaccine strains. Attempted PCR amplification of adventitious tumor-related pathogens, including ALV, REV, and CIAV, showed no products, and enzyme-linked immunosorbent assay results for ALV and indirect fluorescent antibody detection of REV were negative. These results demonstrate the purity of the isolated AH/1807 and DH/18 strains.

### 3.2. Occurrence Rate and Distribution of MD Tumors 

**Figure 2:**
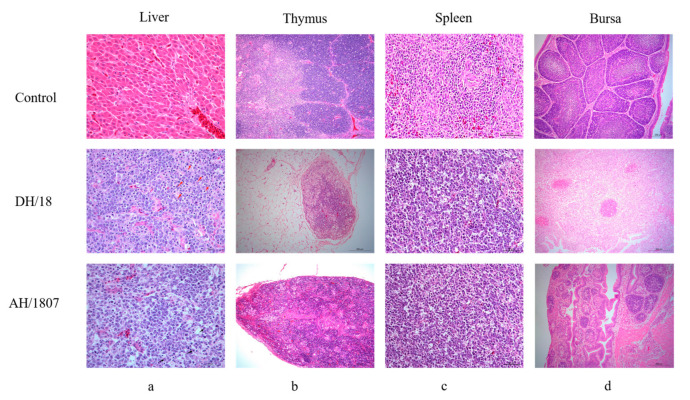
Hematoxylin and eosin-stained histological lesions of collected tissues. Control, AH/1807-, and DH/18-challenged groups were compared for each tissue. (**a**) Liver, DH/18 invasive growth of tumor cells, pyknosis of liver nuclei, and excessive necrosis. AH/1807, a large number of tumor cells infiltrating and proliferating, with frequent pathological mitotic phases. Liver cells are necrotic in large numbers, and normal tissues are rarely visible. Tumor cells are mainly lymphoblastic. Scale bar: 50 µm. (**b**) Thymus, DH/18 atrophy, significant decrease in cortical lymphocytes, proliferation of macrophages, and proliferation of adipose tissue around the thymus. AH/1807 thymus atrophy, massive necrosis, and reduction in cortical lymphocytes, and proliferation of macrophages. Scale bar: 200 µm. (**c**) Spleen, DH/18 local necrosis of the parenchyma with a large number of tumor cells infiltrating, and the tumor cells are mainly lymphoblastic. AH/1807, local necrosis of the parenchyma with a large number of tumor cells infiltrating, most of which are mitotic, and the tumor cells are mainly lymphoblastic. Scale bar: 50 µm. (**d**) Bursa, DH/18 follicle atrophy, multiple necrosis, massive necrosis, reduction in lymphocytes, and mild interstitial hyperplasia. AH/1807 fold atrophy, significant necrosis of follicular lymphocytes, and interstitial hyperplasia. Scale bar: 200 µm.

After observations were recorded regarding their overall appearance, chickens were autopsied and all tumors were examined and counted. The tumor rate was higher in chickens infected with AH/1807 (group 3: 50%) than in those infected with DH/18 (group 7: 33.3%; Table 3); however, both isolates led to the development of visible tumors in organs (Figure 2a,c) and atrophy of the thymus and bursa (Figure 2b,d). Neither chickens in the vaccinated AH/1807-challenged (group 5), nor those in the vaccinated DH/18-challenged (group 9) groups produced tumors.

### 3.3. MD Morbidity and Mortality

One chicken was excluded from group 5, as its death was attributed to a chick quality problem acknowledged by the supplier. All other MD morbidity and mortality data are shown in Table 3. The mortality rate among AH/1807-challenged chickens (group 3: 77.8%) was markedly higher than that among DH/18-challenged chickens (group 7: 50%). However, the incidence of MD after CVI988 immunization was substantially higher in chickens challenged with DH/18 (group 9: 38.9%) than in those challenged with AH/1807 (group 5: 5.9%). Thus, the PI of CVI988 against AH/1807 (group 5: 94.1%) was considerably higher than that against DH/18 (group 9: 61.1%). In addition, mortality in the vaccinated AH/1807-challenged group (group 5: 5.9%) was higher than that in the vaccinated DH/18-challenged group (group 9: 0%). In summary, our results indicate that although infection with DH/18 caused less pathogenic damage in chickens than infection with AH/1807, the vaccine offered lower protection against DH/18 compared with AH/1807.

### 3.4. Survival Analysis

**Figure 3:**
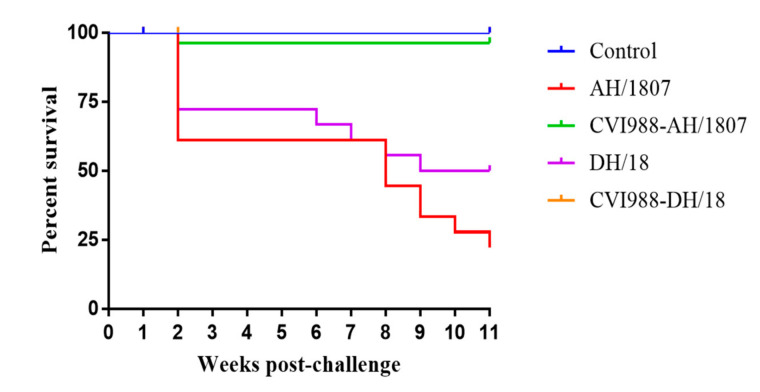
Chicken survival curves for each treatment group. The survival patterns in the AH/1807-challenged and DH/18-challenged groups exhibited effects of infection with two strains. It exhibited significant differences (*p* < 0.05) by log-rank (Mantel–Cox) test.

The earliest observed deaths occurred in the AH/1807- and DH/18-challenged groups (groups 3 and 7, respectively) at 9 dpc. Infection with DH/18 caused programmed death in chickens two weeks earlier than did infection with AH/1807, starting from the sixth week post-challenge. However, AH/1807 caused more severe programmed deaths than DH/18, and these occurrences continued until the end of the experiment. Survival analysis showed that differences were observed in the death patterns of chickens challenged with AH/1807 compared to those challenged with DH/18 (Figure 3).

### 3.5. Developmental Disorders and Immune Organ Damage 

**Figure 4:**
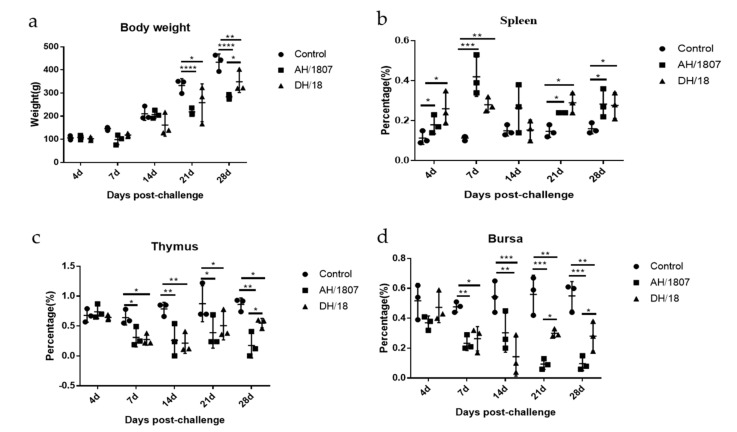
Ratios of immune organ weight to body weight in chickens of each treatment group. The body weights and immune organ indices of MDV strains at 4, 7-, 14-, 21-, and 28-days post challenge were analyzed. (**a**) Body weight of chickens in each group. (**b**) Ratio of spleen weight to body weight in chickens of each group. (**c**) Ratio of thymus weight to body weight in chickens of each group. (**d**) Ratio of bursa weight to body weight in chickens of each group. Two-way ANOVA was performed for significance analysis. The data are shown as mean with standard deviations (SDs). * *p* < 0.05, ** *p* < 0.01, *** *p* < 0.001, **** *p* < 0.0001.

Immune organ damage and developmental disorders are typical symptoms of MD. As such, dynamic changes in immune organ indices were analyzed (groups 2, 4, 6, and 8) (Figure 4a–d). At 21 and 28 dpc, the body weights of chickens in the two challenged groups (groups 2 and 6) were lower than those of chickens in the control group (group 1). Spleens collected from chickens in the two challenged groups exhibited significant swelling compared to those collected from chickens in the control group at all measured time points, except for 14 dpc. Starting from 7 dpc, atrophy was detected in the thymus and bursa of chickens in the challenged groups. AH/1807 induced more atrophy of the thymus than DH/18 at 28 dpc. At 21 and 28 dpc, the bursa of chickens in the AH/1807-challenged group were more atrophied than those of chickens in the DH/18-challenged group. These results indicate that the two strains cause different degrees of damage to the thymus and bursa at some time points. DH/18-challenged chickens exhibited less damage to these organs than AH/1807-challenged chickens during the prophase of infection, implying that DH/18 may be less pathogenic than AH/1807.

**Figure 5:**
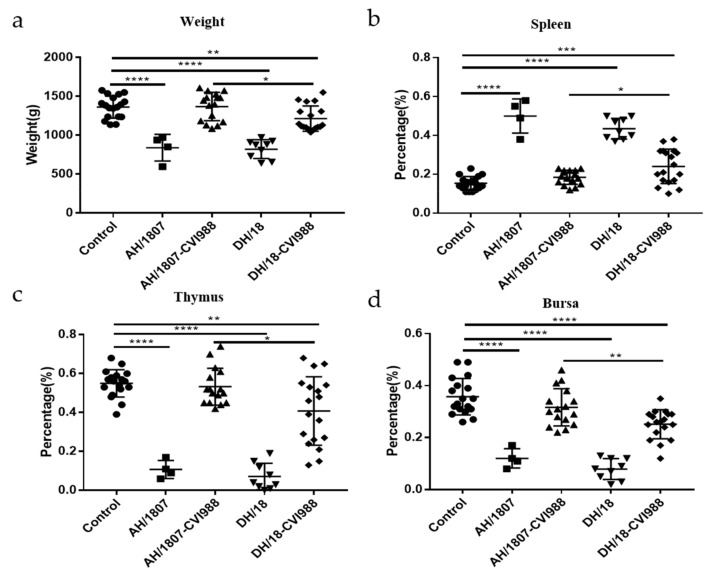
Body weight and ratio of immune organ weight to body weight in control and challenged groups at 72-days post challenge. (**a**) Body weight. (**b**) Ratio of spleen weight to body weight. (**c**) Ratio of thymus weight to body weight. (**d**) Ratio of bursa weight to body weight. T tests were performed for significance analysis. The data are shown as means with standard deviations (SDs). * *p* < 0.05, ** *p* < 0.01, *** *p* < 0.001, **** *p* < 0.0001.

At the end of the experimental period (72 dpc), the body weights and immune organ indices of all surviving chickens in each group (groups 3, 5, 7, and 9) were calculated and analyzed (Figure 5a–d). The average body weight of chickens in the AH/1807- and DH/18-challenged groups was lower than that of chickens in the control group, indicating that both strains can cause severe developmental disorders. The average body weight of chickens in the vaccinated DH/18-challenged group was significantly lower than that of chickens in the control group; however, no significant difference was observed in average body weight between the vaccinated AH/1807-challenged group and the control group. The average body weight of chickens in the vaccinated AH/1807-challenged group was much lower than that of chickens in the vaccinated DH/1807-challenged group. Regarding immune organs, both AH/1807 and DH/18 caused severe atrophy of the thymus and bursa, and both were observed to lead to splenomegaly in some cases. However, among the vaccinated-challenged groups, only the vaccinated DH/18-challenged group exhibited a significant difference in immune organ index compared with the control group. In addition, the degree of damage to the spleen, thymus, and bursa in chickens from the vaccinated DH/18-challenged group was generally more serious than that in chickens from the vaccinated AH/1807-challenged group. Although some variation was observed between individual chickens, these results suggest that the vaccine may be less protective against DH/18 than it is against AH/1807.

### 3.6. Kinetics of Viral Replication In Vivo

**Figure 6:**
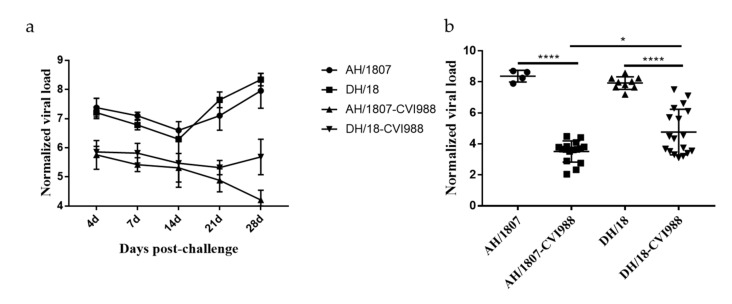
Normalized viral loads in the spleens of chickens from various treatment groups. (**a**) The normalized viral loads from the spleens of three chickens randomly selected from each group at 4-, 7-, 14-, 21-, and 28-days post challenge (dpc). (**b**) The normalized viral loads in the spleens of all surviving chickens in each group at 72 dpc. Normalized viral loads were calculated as the logarithm of the MDV copy number per million cells. T tests were performed for significance analysis. The data are shown as means with standard deviations (SDs). * *p* < 0.05, **** *p* < 0.0001.

Although this qPCR method cannot differentiate the *meq* gene of virulent strains from that of CVI988, the viral load of the vaccine strain is much lower than that of the virulent strain due to the lower replication capacity of the vaccine strain in vivo. For vaccinated-challenged groups, although the total viral load is detected, the viral load of isolates is dominant. We quantified viral genomes including the CVI988 genome, but AH/1807 and DH/18 can be mainly quantified. The ability of the two strains to replicate in vivo was analyzed, and the viral load of the spleen samples was quantified (Figure 6). Dynamic analysis of viral replication in vivo was performed on samples from groups 2 and 6 (Figure 6a). The viral replication titers of the two strains increased at 4 dpc, indicating that they entered the cytolytic infection phase at this point. After that, the replication ability of the two strains decreased, reaching its lowest level at 14 dpc, which indicated that both strains had entered the latent infection period. Subsequently, the replication titer increased substantially as the strains entered the reactivation and tumor transformation phases. A marked difference was observed in replication titer trends between the vaccinated-challenged groups for each strain (groups 4 and 8), with the replication titer of AH/1807 beginning to increase from 21 dpc in vaccinated AH/1807-challenged chickens, while the replication titer of DH/18 decreased substantially from this point in vaccinated DH/18-challenged chickens. The viral loads of surviving chickens were determined at 72 dpc (Figure 6b) from spleen samples obtained from groups 3, 5, 7, and 9. No significant differences were found in terms of viral load between chickens challenged with the two strains. However, viral loads in vaccinated AH/1807-challenged chickens were lower than those in vaccinated DH/18-challenged birds. These results show that the vaccine had a strong inhibitory effect on both strains in vivo, though it was less effective at inhibiting DH/18 than AH/1807.

### 3.7. Expression of IFN-β and IFN-γ mRNA

**Figure 7:**
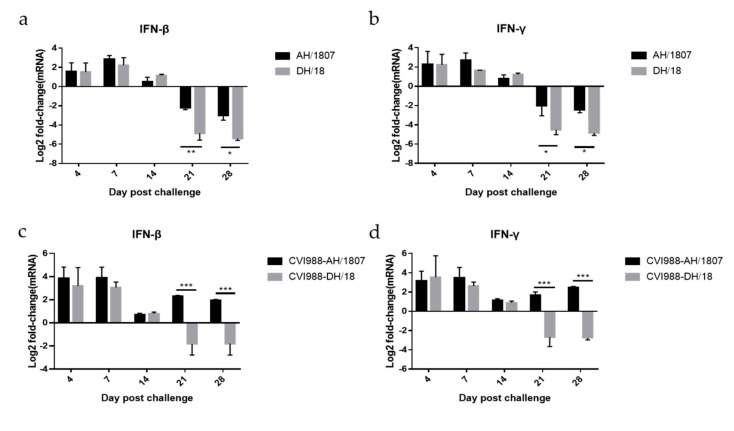
The mRNA expression of IFN-β and IFN-γ in the spleen. Three chickens were randomly selected at each time point (4-, 7-, 14-, 21-, and 28-days post challenge), and the relative expression of interferons (IFNs) was detected using quantitative real-time polymerase chain reaction. Bars above the horizontal line were upregulated and those below were downregulated. Comparing the expression levels of (**a**) IFN-β and (**b**) IFN-γ between AH/1807 and DH/18 groups. Comparing the expression levels of (**c**) IFN-β and (**d**) IFN-γ between CVI988-AH/1807 and CVI988-DH/18 groups. Two-way ANOVA was performed for significance analysis. The data are shown as means with standard deviations (SDs). * *p* < 0.05, ** *p* < 0.01, *** *p* < 0.001.

The mRNA expression of IFN-β and IFN-γ in the spleen was dynamically detected, and RT-qPCR was used to detect expression in five groups of chickens: control (group 1), AH/1807- (group 2), vaccinated AH/1807- (group 4), DH/18- (group 6), and vaccinated DH/18-challenged (group 8) chickens. The expression of IFN-β and IFN-γ in chickens from the four treatment groups relative to those in the control group was calculated and analyzed using logarithms (Figure 7a–d). Expression of IFN-β and IFN-γ was upregulated prior to 14 dpc in AH/1807-challenged chickens (group 2) and DH/18-challenged chickens (group 6) relative to control chickens. However, at 21 and 28 dpc, expression of IFN-β and IFN-γ was downregulated in AH/1807- and DH/18-challenged chickens relative to controls. Additionally, expression of IFN-β and IFN-γ in DH/18-challenged chickens was much lower than that in AH/1807-challenged chickens at 21 and 28 dpc. These results suggest that DH/18 has a stronger immunosuppressive ability than AH/1807. 

Among the vaccinated groups, the vaccinated AH/1807-challenged group exhibited upregulated expression of IFN-β and IFN-γ relative to controls, while the vaccinated DH/18-challenged group exhibited downregulated expression at 21 and 28 dpc. Additionally, substantial differences were observed in IFN-β and IFN-γ expression between the two vaccinated-challenged groups. IFN-β and IFN-γ were upregulated in the vaccinated-challenged groups, relative to the challenged groups, suggesting that vaccination may boost IFN-β and IFN-γ expression. However, infection with DH/18 still caused inhibition of the expression of IFN-β and IFN-γ after vaccination at 21 and 28 dpc, suggesting that DH/18 may have a stronger immunosuppressive ability than AH/1807 after vaccination.

### 3.8. Sequence Alignment and Phylogenetic Analysis

**Figure 8:**
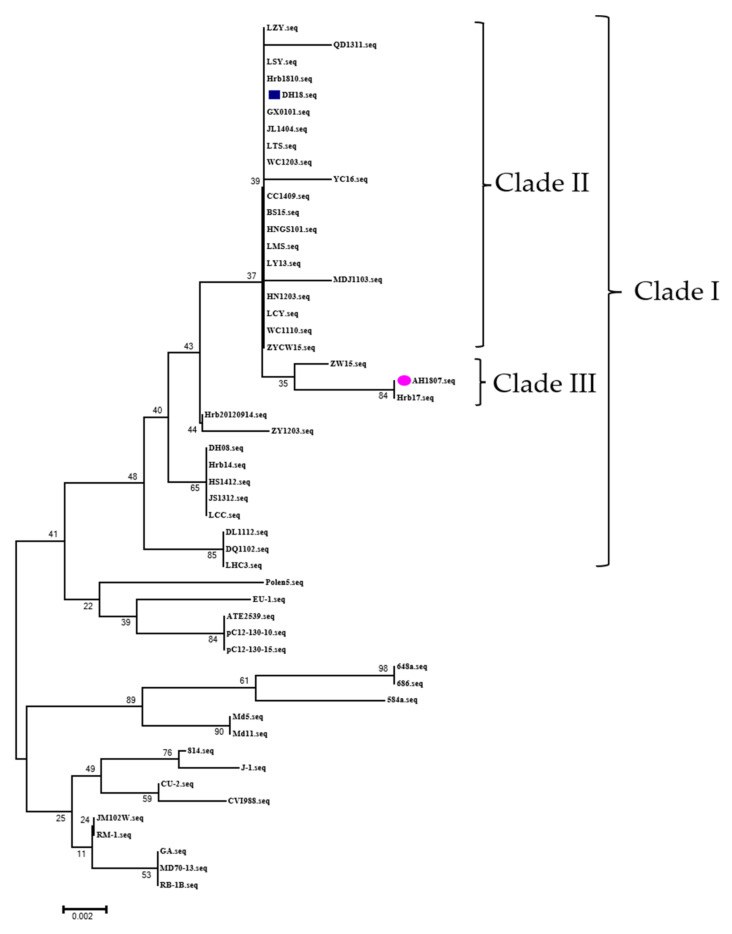
Phylogenetic tree based on *meq* gene complete amino acid sequences of AH/1807 and DH/18 that marked with a colored dot. Other strains were retrieved from GenBank.

The comparison and analysis of the *meq* sequences of the two strains showed that the substitutions of the *meq* amino acid was the characteristic of the epidemic strains in China [27]. Compared with the GA strain, AH/1807 and DH/18 had the same amino acid mutation at position 77 (K to E), 80 (D to Y), 115 (V to A), 139 (T to A), 176 (P to R), and 217 (P to A) (Table 4). However, AH/1807 had mutations at positions 88 (A to T) and 93 (Q to R), while DH/18 had no changes at the same position. The phylogenetic tree analysis based on the *meq* amino acid sequence showed that the two strains were located in clade I with the epidemic strains in China (Figure 8). DH/18 is in a different clade than AH/1807 and it is in clade II with LTS [16] and BS [14].

## 4. Discussion

MD is one of the most important diseases endangering the poultry industry [28]. MD kills chickens directly and can cause infected chickens to develop immunosuppression [29,30]. Despite the widespread use of vaccines in China, MD vaccination failure has occurred in recent years [13]. China’s poultry industry is developed, dense, and extensive, and MDV strains are diverse and evolving [14,25]; thus, there may be a variety of pandemic strains with different pathogenic characteristics present in China. The evolution of MDV strains is the main cause of immune failure of vaccines, and thus studying differences in MDV strains could further reveal the reasons for this immune failure. Here, we isolated two strains that cause different clinical symptoms (AH/1807 and DH/18) from two chicken farms, analyzed their pathogenic characteristics, and evaluated their ability to resist the host’s innate immunity and the immune effects caused by vaccination. 

SPF chickens were artificially infected with one of two strains. We conducted parallel tests in combination with pathogenicity and vaccine immune protection tests. The rates of mortality and tumors caused by AH/1807 were higher than those caused by DH/18. In addition, the damage to immune organs caused by DH/18 was lower than that caused by AH/1807 before 28 dpc. These results indicate that the pathogenicity characteristics of the two strains are very different, with AH/1807 showing higher pathogenicity than DH/18. In the vaccine protection experiment, CVI988 provided substantially different PIs for SPF chickens challenged with AH/1807 and those challenged with DH/18. This finding suggests that DH/18 can break through the vaccine protection of CVI988. The period of MDV infection can be determined by assessing the viral load in the host’s organs [31]. We found that the replication titer was lowest at 14 dpc, which is when both strains were considered to have entered the latent infection state [24]. Subsequently, the replication capacity of DH/18 was higher than that of AH/1807 during both the reactivation and transformation stages. The latent viral load of DH/18 tended to be lower than that of AH/1807; however, this strain had a stronger replication ability during the reactivation and transformation periods. A low viral load may have allowed DH/18 to better evade the host immune response and replicate in large quantities, which may partially explain its ability to break through immune protection. 

Mutations in the *meq* gene can affect the virulence and vaccine resistance of MDV strains [32]. We conducted a comparative analysis of the tumorigenic gene *meq*, and both strains had the characteristics of the epidemic strains in China [27]. Additionally, the disruption of the proline repeats of the two strains enhanced virulence [33]. Although AH/1807 did not break through the immune protection of the CVI988 vaccine, the tumor incidence of AH/1807 is higher than DH/18. Mutations in *meq* can affect tumor incidence and vaccinal protection [32]. Compared with DH/18, the *meq* of AH/1807 had mutations at positions 88 and 93, which may be related to the enhanced tumor incidence and decreased vaccine resistance, which needs to be verified with further research. The results of phylogenetic tree analysis showed that AH/1807 was located in clade III, which was different from DH/18. DH/18 is located in the same clade as LTS and BS/15. LTS caused very low mortality (23.1%) in unvaccinated chickens, but CVI988 did not provide good immune protection against it (PI:66.7%) [16]. BS and Md5 caused similar mortality in unvaccinated chickens, but BS was able to break through the immune protection of the CVI988 vaccine while Md5 could not [14].

DH/18 shared characteristics with them. These strains are currently circulating in China, and the strains are diverse [34,35]. It is difficult to define the phenotypes of strains according to the previous criteria due to the divergence of virulence and vaccine resistance.

The evasion of the innate immune response is essential for herpesviruses to establish infection, latency, and lifelong persistence in the host [36]. IFNs are cytokines that induce the upregulation of cellular antiviral states and are major components of the innate antiviral host defense. While type I IFNs (IFN-α and IFN-β) are secreted by many different cell types, type II (IFN-γ) is predominantly produced by T helper 1 cells and natural killer cells [37]. During the initial stages of MDV infection in this study, chickens were in an antiviral state, producing IFN-β and IFN-γ and thereby inhibiting MDV replication. At 14 dpc, the cell replication titer for both strains reached its lowest point, and MDV entered the latent period. Subsequently, MDV infection inhibited the expression of IFN-β and IFN-γ, resulting in enhanced MDV replication ability and enhanced lesions. IFN-γ can inhibit MDV replication in a dose-dependent manner, with higher doses producing stronger inhibitory effects [38]. Some studies have suggested that MDV could inhibit the expression of IFN-β, a process which is involved in MDV-induced host immunosuppression and contributes to the escape of MDV from host immunity [29,39]. Another study reported that MDV could inhibit IFN-β production in the late phase of infection [30]. We found that the inhibitory effect of DH /18 on IFN-β and IFN-γ was stronger than that of AH/1807, resulting in the replication capacity of DH/18 tending to be higher than that of AH/1807.

Different MDV strains can have different effects on IFN expression, a factor which may be related to their diversity in terms of virulence and pathogenicity [40]. In our study, the vaccinated-challenged group exhibited increased expression of IFN-γ compared with the challenged group in the hosts. We also verified that vaccination can increase the expression of IFN-β. The expression of IFN-β and IFN-γ can be upregulated in the spleen by only inoculating with CVI988 [41,42]. Vaccination inhibited MDV replication by increasing the expression of IFN-β and IFN-γ, which may play important roles in immune protection, as IFN-γ is a key factor in MD vaccine-induced protection [43]. Vaccination can also increase IFN-γ expression, which can improve immune protection after MDV infection [44]. However, this was not the case during DH/18 infection. Even after vaccination, DH/18 inhibited the expression of IFN-β and IFN-γ, leading to an increased number of MDV replications. This may be an important factor underlying this strain’s ability to break through immune protection. Although some previous studies have shown that the *meq* and *RLORF4* genes of MDV can inhibit IFN-β expression [29,39], more studies are needed to explore the mechanism of how MDV inhibits the expression of IFNs. In this study, we did not explore how MDV strains inhibited IFNs, nor did we explore the mechanisms by which different strains have different effects on IFNs.

This study revealed significant differences between these two MDV strains in terms of pathogenesis and vaccine resistance Although AH/1807 had higher pathogenicity than DH/18, DH/18 was more resistant to the CVI988 vaccine. This may be because DH/18 has stronger immunosuppressive abilities and replication features, characteristics that should be considered for the future control of MD. The epidemic strains of MDV in China are constantly evolving and are able to break through the immune protection provided by vaccines. In this study, we elucidated the factors that may contribute to this failure of immunity. Ineffective vaccines have resulted in many difficulties in the precise prevention and control of MD and could be the reason for the continuous occurrence of MD in China in recent years. Therefore, a broader and more effective vaccine against MDV strains needs to be developed.

## Figures and Tables

**Table 1:** Vaccinated and challenged experimental groups used in this study.

Group	No. of Chickens	Vaccine	Challenge
1	36	-	-
2	18	-	AH/1807
3	18	-	AH/1807
4	18	CVI988	AH/1807
5	18	CVI988	AH/1807
6	18	-	DH/18
7	18	-	DH/18
8	18	CVI988	DH/18
9	18	CVI988	DH/18

**Table 2:** Primer and probe sequences used for quantitative real-time polymerase chain reaction.

Gene	Type	Sequence (5′–3′)	Amplicon Size (bp)
*meq*	Forward Reverse Probe	GGAGCCGGAGAGGCTTTATG ATCTGGCCCGAATACAAGGAA CGTCTTACCGAGGATCCCGAACAGG	69
*ovo*	Forward Reverse Probe	CACTGCCACTGGGCTCTGT GCAATGGCAATAAACCTCCAA AGTCTGGAGAAGTCTGTGCAGCCTCCA	71
*IFN-γ*	Forward Reverse Probe	TACTGAGCCAGATTGTTTCGAT TCACCTTCTTCACGCCAT AAGTCAAAGCCGCACATCAAACAC	132
*IFN-β*	Forward Reverse Probe	CACAACAAGACGTGACTTTTCCATTT AAGCATGTTGAAGAGGTGTTGGAG AGGACAAGAAGCAAGCAGCCATCACCACC	110
*28S*	Forward Reverse Probe	GGCGAAGCCAGAGGAAACT GACGACCGATTTGCACGTC AGGACCGCTACGGACCTCCACCA	62

Abbreviations: ovo, ovotransferrin; IFN, interferon; 28S, a housekeeping gene.

**Table 3:** Morbidity, mortality, and tumor rates of Marek’s disease (MD) by group.

Group	Vaccine	Challenge	MD Incidence	PI	Mortality	Tumor Incidence	Time (dpc)
1	-	-	-	-	-	-	
3	-	AH/1807	18/18(100%)	-	14/18(77.8%)	9/18(50%)	72
5	CVI988	AH/1807	1/17(5.9%)	94.1	1/17(5.9%)	0%	72
7	-	DH/18	18/18(100%)	-	9/18(50%)	6/18(33.3%)	72
9	CVI988	DH/18	7/18(38.9%)	61.1	0/18(0%)	0%	72

Abbreviations: dpc, days post challenge, PI, protective index, MD incidence: T-cell lymphomas, solid visceral tumors, immune organ atrophy, neurological disorders, death, or health complications.

**Table 4:** Amino acid substitutions in *meq* oncoprotein of the two isolates.

Strains	77	80	88	93	115	139	176	217
GA	K	D	A	Q	V	T	P	P
AH/1807	E	Y	T	R	A	A	R	A
DH/18	E	Y	A	Q	A	A	R	A

## Data Availability

The raw data supporting the conclusions of this article are available from the corresponding author upon reasonable request.

## Supplementary Materials

The following supporting information can be downloaded at: https://www.mdpi.com/article/10.3390/v15040945/s1, Table S1: *meq* gene sequencing results with AH/1807 and DH/18.

## References

[1] Gimeno I.M., Cortes A.L., Faiz N.M., Hernandez-Ortiz B.A., Guy J.S., Hunt H.D., Silva R.F. (2015). Evaluation of the Protection Efficacy of a Serotype 1 Marek’s Disease Virus-Vectored Bivalent Vaccine Against Infectious Laryngotracheitis and Marek’s Disease. Avian Dis..

[2] Churchill A.E., Biggs P.M. (1967). Agent of Marek’s Disease in Tissue Culture. Nature.

[3] Nair V. (2005). Evolution of Marek’s disease—A paradigm for incessant race between the pathogen and the host. Vet. J..

[4] Witter R.L., Calnek B.W., Buscaglia C., Gimeno I., Schat K.A. (2005). Classification of Marek’s disease viruses according to pathotype: Philosophy and methodology. Avian Pathol..

[5] Teng M., Zheng L.-P., Li H.-Z., Ma S.-M., Zhu Z.-J., Chai S.-J., Yao Y., Nair V., Zhang G.-P., Luo J. (2022). Pathogenicity and Pathotype Analysis of Henan Isolates of Marek’s Disease Virus Reveal Long-Term Circulation of Highly Virulent MDV Variant in China. Viruses.

[6] Gimeno I.M. (2008). Marek’s disease vaccines: A solution for today but a worry for tomorrow?. Vaccine.

[7] Morrow C., Fehler F., Davison F., Nair V. (2004). 5-Marek’s disease: A worldwide problem. Marek’s Disease.

[8] Davison F., Nair V. (2005). Use of Marek’s disease vaccines: Could they be driving the virus to increasing virulence?. Expert Rev. Vaccines.

[9] Kennedy D.A., Read A.F. (2018). Why the evolution of vaccine resistance is less of a concern than the evolution of drug resistance. Proc. Natl. Acad. Sci. USA.

[10] Zhang Y., Lan X., Wang Y., Lin Y., Yu Z., Guo R., Li K., Cui H., Qi X., Wang Y. (2022). Emerging natural recombinant Marek’s disease virus between vaccine and virulence strains and their pathogenicity. Transbound. Emerg. Dis..

[11] Yu Z., Zhang Y., Lan X., Wang Y., Zhang F., Gao Y., Li K., Gao L., Pan Q., Qi X. (2019). Natural co-infection with two virulent wild strains of Marek’s disease virus in a commercial layer flock. Vet. Microbiol..

[12] Li K., Yu Z., Lan X., Wang Y., Qi X., Cui H., Gao L., Wang X., Zhang Y., Gao Y. (2022). Complete genome analysis reveals evolutionary history and temporal dynamics of Marek’s disease virus. Front. Microbiol..

[13] Li H., Ge Z., Luo Q., Fu Q., Chen R. (2022). A highly pathogenic Marek’s disease virus isolate from chickens immunized with a bivalent vaccine in China. Arch. Virol..

[14] Sun G.-R., Zhang Y.-P., Lv H.-C., Zhou L.-Y., Cui H.-Y., Gao Y.-L., Qi X.-L., Wang Y.-Q., Li K., Gao L. (2017). A Chinese Variant Marek’s Disease Virus Strain with Divergence between Virulence and Vaccine Resistance. Viruses.

[15] Teng L.-Q., Wei P., Song Z.-B., Yang N.-L. (2009). Evaluation of the pathogenicity of a field isolate of Marek’s disease virus integrated with retroviral long terminal repeat sequence. Bing du xue bao = Chin. J. Virol..

[16] Zhang Y.-P., Li Z.-J., Bao K.-Y., Lv H.-C., Gao Y.-L., Gao H.-L., Qi X.-L., Cui H.-Y., Wang Y.-Q., Ren X.-G. (2015). Pathogenic characteristics of Marek’s disease virus field strains prevalent in China and the effectiveness of existing vaccines against them. Vet. Microbiol..

[17] Lv H.C., Zhang Y.P., Sun G.R., Gao Y.L., Li Z.J., Zheng H.W., Bao K.Y., Wang X.M., Liu C.J. (2016). Assessments of a PCR method for detection and identification of virulent Marek’s disease virus and vaccine strain for Marek’s disease diagnosis. Chin. J. Prev. Vet. Med..

[18] Li H., Wang P., Lin L., Shi M., Gu Z., Huang T., Mo M., Wei T., Zhang H., Wei P. (2018). The emergence of the infection of subgroup J avian leucosis virus escalated the tumour incidence in commercial Yellow chickens in Southern China in recent years. Transbound. Emerg. Dis..

[19] Nishitha Y., Priyanka E., Krishna S.V., Kannaki T.R. (2021). Co-infection of Marek’s disease virus with different oncogenic immunosuppressive viruses in chicken flocks. Virusdisease.

[20] Haridy M., Goryo M., Sasaki J., Okada K. (2009). Pathological and immunohistochemical study of chickens with co-infection of Marek’s disease virus and chicken anaemia virus. Avian Pathol..

[21] Gopal S., Manoharan P., Kathaperumal K., Chidambaram B., Divya K.C. (2012). Differential Detection of Avian Oncogenic Viruses in Poultry Layer Farms and Turkeys by Use of Multiplex PCR. J. Clin. Microbiol..

[22] Qin L.T., Gao Y.L., Pan W., Deng X.Y., Sun F.F., Li K., Qi X.-G.L., Gao H.L., Liu C.N., Wang X.M. (2010). Investigation of co-infection of ALV-J with REV, MDV, CAV in layer chicken flocks in some regions of China. Chin. J. Prev. Vet. Med..

[23] Witter R.L., Kreager K.S. (2004). Serotype 1 viruses modified by backpassage or insertional mutagenesis: Approaching the threshold of vaccine efficacy in Marek’s disease. Avian Dis..

[24] Baigent S.J., Petherbridge L.J., Howes K., Smith L.P., Currie R.J., Nair V.K. (2005). Absolute quantitation of Marek’s disease virus genome copy number in chicken feather and lymphocyte samples using real-time PCR. J. Virol. Methods.

[25] Gong Z., Zhang L., Wang J., Chen L., Shan H., Wang Z., Ma H. (2013). Isolation and analysis of a very virulent Marek’s disease virus strain in China. Virol. J..

[26] Sharma J.M., Burmester B.R. (1982). Resistance to Marek’s disease at hatching in chickens vaccinated as embryos with the turkey herpesvirus. Avian Dis..

[27] Zhang Y.-P., Liu C.-J., Zhang F., Shi W., Li J. (2011). Sequence analysis of the Meq gene in the predominant Marek’s disease virus strains isolated in China during 2006–2008. Virus Genes.

[28] Deng Q., Shi M., Li Q., Wang P., Li M., Wang W., Gao Y., Li H., Lin L., Huang T. (2020). Analysis of the evolution and transmission dynamics of the field MDV in China during the years 1995–2020, indicating the emergence of a unique cluster with the molecular characteristics of vv+ MDV that has become endemic in southern China. Transbound. Emerg. Dis..

[29] Li K., Liu Y., Xu Z., Zhang Y., Luo D., Gao Y., Qian Y., Bao C., Liu C., Zhang Y. (2019). Avian oncogenic herpesvirus antagonizes the cGAS-STING DNA-sensing pathway to mediate immune evasion. PLoS Pathog..

[30] Du X., Zhou D., Zhou J., Xue J., Wang G., Cheng Z. (2022). Marek’s disease virus serine/threonine kinase Us3 facilitates viral replication by targeting IRF7 to block IFN-β production. Veter-Microbiol..

[31] Couteaudier M., Denesvre C. (2014). Marek’s disease virus and skin interactions. Vet. Res..

[32] Conradie A.M., Bertzbach L.D., Trimpert J., Patria J.N., Murata S., Parcells M.S., Kaufer B.B. (2020). Distinct polymorphisms in a single herpesvirus gene are capable of enhancing virulence and mediating vaccinal resistance. PLoS Pathog..

[33] Spatz S.J., Petherbridge L., Zhao Y., Nair V. (2007). Comparative full-length sequence analysis of oncogenic and vaccine (Rispens) strains of Marek’s disease virus. J. Gen. Virol..

[34] Cui N., Su S., Sun P., Zhang Y., Han N., Cui Z. (2016). Isolation and pathogenic analysis of virulent Marek’s disease virus field strain in China. Poult. Sci..

[35] Zhang Y.-P., Lv H.-C., Bao K.-Y., Gao Y.-L., Gao H.-L., Qi X.-L., Cui H.-Y., Wang Y.-Q., Li K., Gao L. (2015). Molecular and pathogenicity characterization of Gallid herpesvirus 2 newly isolated in China from 2009 to 2013. Virus Genes.

[36] Beachboard D.C., Horner S.M. (2016). Innate immune evasion strategies of DNA and RNA viruses. Curr. Opin. Microbiol..

[37] Lee A.J., Ashkar A.A. (2018). The Dual Nature of Type I and Type II Interferons. Front. Immunol..

[38] Bertzbach L.D., Harlin O., Härtle S., Fehler F., Vychodil T., Kaufer B.B., Kaspers B. (2019). IFNα and IFNγ Impede Marek’s Disease Progression. Viruses.

[39] Liu Y., Gao L., Xu Z., Luo D., Zhang Y., Gao Y., Liu C., Zhang Y., Qi X., Cui H. (2019). Marek’s Disease Virus RLORF4 Inhibits Type I Interferon Production by Antagonizing NF-κB Activation. J. Virol..

[40] Sun G.-R., Zhou L.-Y., Zhang Y.-P., Zhang F., Yu Z.-H., Pan Q., Gao L., Li K., Wang Y.-Q., Cui H.-Y. (2019). Differential expression of type I interferon mRNA and protein levels induced by virulent Marek’s disease virus infection in chickens. Vet. Immunol. Immunopathol..

[41] Gimeno I.M., Glaize A., Cortes A.L. (2018). Effect of Marek’s disease vaccines on interferon and toll like receptors when administered in ovo. Vet. Immunol. Immunopathol..

[42] Jin H., Kong Z., Mehboob A., Jiang B., Xu J., Cai Y., Liu W., Hong J., Li Y. (2020). Transcriptional Profiles Associated with Marek’s Disease Virus in Bursa and Spleen Lymphocytes Reveal Contrasting Immune Responses during Early Cytolytic Infection. Viruses.

[43] Kano R., Konnai S., Onuma M., Ohashi K. (2009). Cytokine profiles in chickens infected with virulent and avirulent Marek’s disease viruses: Interferon-gamma is a key factor in the protection of Marek’s disease by vaccination. Microbiol. Immunol..

[44] Boodhoo N., Behboudi S. (2022). Differential Virus-Specific IFN-Gamma Producing T Cell Responses to Marek’s Disease Virus in Chickens With B19 and B21 MHC Haplotypes. Front. Immunol..

