# Supplementary material for: Differences in Pathogenicity and Vaccine Resistance Discovered between Two Epidemic Strains of Marek’s Disease Virus in China
Source: Viruses. 2023 Apr 11;15(4):945. doi: 10.3390/v15040945 (PMC10145439; doi:10.3390/v15040945)
Supplement: Supplementary file 1 [file viruses-15-00945-s001.zip › viruses-2308378-supplementary.pdf]

Supplementary Table S1: *meq* gene sequencing results with AH/1807 and DH/18

Stain	Sequence of meq
AH/1807	ATGTCTCAGGAGCCAGAGCCGGGCGCTATGCCCTACAGTCCCGCTGAC<br>GATCCGTCCCCCCTCGATCTTTCTCTCGGGTCGACTTCGAGACGGAAAA<br>AAAGGAAAAGTCACGACATCCCCAACAGCCCCTCCAAACACCCCTTC<br>CCTGACGGCCTATCTGAGGAGGAGAAACAGAAGCTGGAAAGGAGGA<br>GAAAAAGGAATCGTGACGCCGCTCGGAGAAGACGCAGGGAGCAGAC<br>GTACTATGTAGACAAACTCCATGAAACATGTGAAGAGCTGCGGAGGGC<br>CAATGAACACCTACGTAAGGAAATTCGAGATCTAAGGACTGAGTGCAC<br>GTCCCTGCGTGCACAGTTGGCTTGTCATGAGCCAGTTTGCCCTATGGCG<br>GTACCCCTAACGGTGACCCTTGGACTGCTTACCGCCCCGCACGATCCC<br>GTTCCTGAACCTCCCATTTGCACTCCTCCACCTCCCTCACCGGATGAAC<br>CTAACGCTCCACATTGCTCCGGTTCCCAACCTCCTATCTGTACCCCCCGT<br>CCTCCCGATACGGAGGAACTTTGCGCCCAGCTCTGCTCGACCCCACCA<br>CCTCCCATCTCTACTCCCCATATTATCTACGCTCCGGGGCCTTCCCCCCT<br>CCAACCTCCTATCTGTACCCCCGCTCCTCCCGATGCGGAGGAGCTTTGC<br>GCCCAGCTCTGCTCGACCCCACCACCTCCCATCTGTACTCCCCATTCCC<br>TCTTCTGCCCTCCCCAGCCTCCATCTCCGGAGGGCATCTTCCCTGCATTG<br>TGTCCTGTTACCGAGCCGTGTACCCCTCCATCGCCGGGGACGGTTTACG<br>CTCAGCTTTGTCCTGTTGGCCAGGCTCCCCTTTTTACCCCATCTCCCCCA<br>CATCCGGCTCCGGAGCCGGAGAGGCTTTATGCTCGTCTTACCGAGGATC<br>CCGAACAGGATTCCTTGTATTCGGGCCAGATTTATATTCAGTTTCCCTCG<br>GATACTCAGTCTACGGTATGGTGGTTTCCAGGTGACGGGAGACCCTGA
DH/18	ATGTCTCAGGAGCCAGAGCCGGGCGCTATGCCCTACAGTCCCGCTGAC<br>GATCCGTCCCCCCTCGATCTTTCTCTCGGGTCGACTTCGAGACGGAAAA<br>AAAGGAAAAGTCACGACATCCCCAACAGCCCCTCCAAACACCCCTTC<br>CCTGACGGCCTATCTGAGGAGGAGAAACAGAAGCTGGAAAGGAGGA<br>GAAAAAGGAATCGTGACGCCGCTCGGAGAAGACGCAGGGAGCAGAC<br>GTACTATGTAGACAAACTCCATGAAGCATGTGAAGAGCTGCAGAGGGC<br>CAATGAACACCTACGTAAGGAAATTCGAGATCTAAGGACTGAGTGCAC<br>GTCCCTGCGTGCACAGTTGGCTTGTCATGAGCCAGTTTGCCCTATGGCG<br>GTACCCCTAACGGTGACCCTTGGACTGCTTACCGCCCCGCACGATCCC<br>GTTCCTGAACCTCCCATTTGCACTCCTCCACCTCCCTCACCGGATGAAC<br>CTAACGCTCCACATTGCTCCGGTTCCCAACCTCCTATCTGTACCCCCCGT<br>CCTCCCGATACGGAGGAACTTTGCGCCCAGCTCTGCTCGACCCCACCA<br>CCTCCCATCTCTACTCCCCATATTATCTACGCTCCGGGGCCTTCCCCCCT<br>CCAACCTCCTATCTGTACCCCCGCTCCTCCCGATGCGGAGGAGCTTTGC<br>GCCCAGCTCTGCTCGACCCCACCACCTCCCATCTGTACTCCCCATTCCC<br>TCTTCTGCCCTCCCCAGCCTCCATCTCCGGAGGGCATCTTCCCTGCATTG<br>TGTCCTGTTACCGAGCCGTGTACCCCTCCATCGCCGGGGACGGTTTACG<br>CTCAGCTTTGTCCTGTTGGCCAGGCTCCCCTTTTTACCCCATCTCCCCCA<br>CATCCGGCTCCGGAGCCGGAGAGGCTTTATGCTCGTCTTACCGAGGATC<br>CCGAACAGGATTCCTTGTATTCGGGCCAGATTTATATT

	CAGTTTCCCTCGGATACTCAGTCTACGGTCTGGTGGTTTCCAGGTGACG GGAGACCCTGA
